# Validation of mortality risk scores after esophagectomy

**DOI:** 10.1007/s00432-024-06074-w

**Published:** 2025-01-28

**Authors:** Sabine Schiefer, Nerma Crnovrsanin, Ingmar F. Rompen, Nicolas Jorek, Mohammed Al-Saeedi, Thomas Schmidt, Henrik Nienhüser, Leila Sisic

**Affiliations:** 1 Department of General, Visceral and Transplantation Surgery, University Hospital Heidelberg, Heidelberg, Germany; 2 Department of General, Visceral and Cancer Surgery, University Hospital of Cologne, Cologne, Germany

**Keywords:** Esophagus cancer, Risk assessment, Mortality risk score

## Abstract

**Purpose:**

Oncological esophagectomy is the mainstay in esophageal cancer treatment, but perioperative mortality remains a significant concern. Various scoring systems exist to identify patients at high risk for postoperative complications and death. In the following, we aim to evaluate and compare these different scoring systems.

**Methods:**

We analyzed data from 714 patients who underwent esophagectomy between 2002 and 2021. Each patient’s risk was calculated using three models: the International Esodata Study Group (IESG) 90-day mortality risk prediction, the Steyerberg 30-day mortality score, and the Fuchs et al. preoperative in-hospital mortality score (Fuchs score). The diagnostic performance of these models was assessed using the area under the receiver operating characteristic (ROC) curves.

**Results:**

Of the 714 patients, the majority (87.67%) underwent abdomino-thoracic esophagectomy with intrathoracic anastomosis. The IESG score classified 52.1% as very low, 26.6% low, 17.5% middle, 2.8% high, and 1% as very high risk, while the Fuchs score identified 94.5% as low-risk and 5.5% as high-risk patients. Mortality rates were 6.9% at 90 days, 3.4% at 30 days, and 6.7% in-hospital. The area under the ROC curve was 0.634 (95%CI: 0.557–0.712) for the IESG model, 0.637 (95%CI: 0.526–0.747) for the Steyerberg score, and 0.686 (95%CI: 0.611–0.760) for the Fuchs score.

**Conclusions:**

Existing risk score systems provide a possibility for preoperative risk stratification, particularly for identifying high-risk patients. However, due to their limited predictive ability, improvements are needed to apply these strategies effectively in clinical practice.

## Background

544,000 people died of esophageal cancer in 2020 worldwide, which makes it the sixth leading cause of cancer-related deaths (Sung et al. 2021). Multimodal treatment strategies have vastly improved the survival of esophageal cancer patients over the past decades, and new therapeutic approaches, such as immunotherapy, are on the rise. However, esophagectomy is still the mainstay of curative treatment for esophageal cancer patients. Even if postoperative morbidity and mortality rates have declined over the last decades due to advances in operation techniques such as minimally invasive approaches, improved intensive care unit treatment, and improved management of complications, postoperative mortality still ranges between 4 and 10% (Enzinger and Mayer 2003; Siegel et al. 2020).

Besides hospital volume, mortality rates depend mainly on the patient’s individual risk profile (Anderson et al. 2011; Ghaferi et al. 2011). For surgeons and patients, the main question is who should be treated by surgery and for whom other treatment options should be considered since mortality risk is too high. Moreover, patients with an increased risk profile should be monitored more closely to identify complications early and reduce mortality through early interventions, such as prophylactic endosponge placement (Schniewind et al. 2013).

Various risk factors for increased mortality have been investigated (van Kooten et al. 2022), and different risk scores have been established (Fuchs et al. 2017; Liu et al. 2000; Ra et al. 2008; Steyerberg et al. 2006; Tekkis et al. 2004; Zhang et al. 1992).

Recently, the International Esodata Study Group (IESG) established a 90-day mortality risk prediction model based on the data of 8403 patients from 39 centers in 19 countries (D’Journo et al. 2021). This prediction model consisting of ten different parameters allows the surgeon to calculate a more individual patient’s risk score than previous score systems. However, validation of this score in a single-center high-volume setting and comparison to previous models is absent in current literature.

Therefore, the aim of this study is to evaluate this score in a large high-volume center cohort and compare its predictive value for postoperative mortality with two other existing scores by Steyerberg and Fuchs (Fuchs et al. 2017; Steyerberg et al. 2006).

## Methods

### Database search

A prospectively maintained database of patients undergoing esophagectomy for upper gastrointestinal tract cancer at the University Hospital Heidelberg, Germany, from January 2002 until December 2021 was used for the analysis. Patients undergoing esophagectomy due to perforations were excluded.

This study complies with the Declaration of Helsinki. Ethical approval was obtained from the Ethics Committee of the Medical Faculty of Heidelberg (S-635/2013), and the patients gave informed written consent in accordance with the local ethics committee’s vote. Mortality rates were defined from the operation to the patients’ death. We followed the TRIPOD guidelines for score validations.

### Scores/Risk assessment

The following parameters necessary for risk score calculation were gathered from the electronic patient files: age, body-mass-index, gender, ECOG, comorbidities, neoadjuvant treatment, hospital volume, tumor histology, and surgical approach. The individual risk score for each scoring system (IESG, Steyerberg, Fuchs) was calculated for all patients.

### Statistical analysis

The areas under the receiver operating characteristic (ROC) curves were used to estimate the diagnostic performance of the different risk scores.

An AUC of 0.5 predicts no discriminative ability, while an AUC of 1 indicates perfect discrimination with 100% sensitivity and 100% specificity. Prediction models with an AUC of 0.8 and above have been labeled as very good to excellent, those with an AUC between 0.7 and 0.8. as moderate, and 0.6 to 0.7 as providing low discrimination.

All analyses were performed using SPSS Statistics^®^, version 28.0.1.0, and RStudio, version 1.3.959.

## Results

A total of 758 patients were identified; data from 714 patients were available to calculate all three risk scores.

The majority of the patients were male (82.2%). The median age was 61.35 (28–85).

626 (87.67%) patients underwent abdomino-thoracic esophagectomy (Ivor-Lewis procedure), 42 (5.88%) underwent abdomino-thoracic esophagectomy with cervical anastomosis, 23 (3.22%) underwent transhiatal esophagectomy with cervical anastomosis, 17 (2.38%) underwent esophago-gastrectomy and 6 (0.84%) underwent other types of resection. 496 (69.47%) patients underwent neoadjuvant treatment, mainly chemotherapy (45.93%), 19.6% received radiochemotherapy, and 0.98% radiotherapy only.

Hospital volume during the period was 22–69 esophagectomies per year.

In our cohort, the observed mortality rates were as follows: 30-day mortality was 3.4%, 90-day mortality was 6.86%, and in-hospital mortality was 6.72%.

### IESG score

According to the distribution of points for the particular risk factors of the IESG score across our cohort (Table 1), patients reached between − 6 and 4 points (Table 2A).

365 patients (51.25%) had very low (1 to 5 points), 191 (26.75%) had low (0 points), 129 (18.1%) had medium (-2 to -1 points), 20 (2.8%) had high (-4 to -3 points), and 8 (1.1%) had very high risk (-5 to -10 points) for postoperative mortality within 90 days (Table 2B). The 90-day mortality in our cohort was 6.86%. Table 2A + B demonstrates the mortality for each risk group.

**Table 1:** Score system and results for IESG and Heidelberg data

		Points	IESG data		Heidelberg data	
			*n*	%	*n*	%
Age	≤ 40	0	170	2.0	16	2.2
	41–50	1	658	7.8	74	10.4
	51–60	1	2076	24.7	221	31.0
	61–70	0	3357	40.0	283	39.6
	71–80	0	1920	22.8	110	15.4
	> 80	-3	222	2.5	10	1.4
BMI	< 18.5	-3	445	5.3	21	2.9
	18.5–24.9	0	3386	40.3	303	42.4
	25-29.9	1	2966	35.3	291	40.8
	≥ 30	1	1606	19.1	99	13.9
Gender	Male	0	6641	79.0	587	82.2
	Female	2	1762	21.0	127	17.8
ECOG	0	0	4205	50.0	448	62.8
	1	-1	3753	44.7	263	36.8
	2	-2	379	4.5	3	0.4
	3	-4	66	0.8	0	0
Comorbidities	Myocardial infarction	-2	442	5.3	27	3.8
	Connective tissue disease	-3	71	0.8	4	0.6
	Peripheral vascular disease	-2	433	5.2	5	0.7
	Liver disease moderate/severe	-5	136	1.6	2	0.3
Neoadjuvant	none	0	1927	23,0	218	30.6
treatment	dCRT	-2	154	1.7	5	0.7
	CTX	0	2393	28.5	328	45.9
	RCTX	-1	3916	46.6	163	22.8
Hospital volume	0-45.9	0	2169	25.8	488	68.3
	46-71.9	0	1975	23.5	226	31.7
	71.9-108.6	0	2355	28.0	0	0
	> 108.6	1	1904	22.7	0	0

Abbreviation: BMI: body mass index; ECOG: Eastern Cooperative Oncology Group; dCRT: definitive chemoradiotherapy; CTX: chemotherapy; RCTX: radiochemotherapy; IESG: International Esodata Study Group

**Table 2:** Results according to risk groups (A) IESG and Heidelberg data, (B) Simplified risk groups and Heidelberg data

A							
Score	Expected mortality	IESG development		IESG validation		Heidelberg data	
		Deceased/total	%	Deceased/total	%	Deceased/total	%
-10	43.2%	1/1	100.0	0/0	0.0	0/0	0.0
-9	35.8%	0/2	0.0	0/0	0.0	0/0	0.0
-8	29.0%	2/2	100.0	1/4	25.0	0/0	0.0
-7	23.1%	4/11	36.4	2/10	20.0	0/0	0.0
-6	18.0%	0/17	0.0	6/23	26.1	1/3	33.3
-5	13.9%	10/60	16.7	4/54	7.4	0/4	0.0
-4	10.6%	12/133	9.0	7/131	5.3	1/5	20.0
-3	8.0%	22/245	9.0	24/239	10.0	3/15	20.0
-2	6.0%	32/421	7.6	19/386	4.9	3/33	9.1
-1	4.4%	44/887	5.0	46/860	5.3	10/92	10.9
0	3.3%	32/1058	3.0	31/1173	2.6	14/190	7.4
1	2.4%	19/826	2.3	16/810	2.0	14/203	6.9
2	1.8%	5/347	1.4	7/383	1.8	1/120	0.8
3	1.3%	1/132	0.8	4/132	3.0	1/38	2.6
4	1.0%	0/26	0.0	2/23	8.7	1/11	9.1
5	0.7%	0/4	0.0	0/2	0.0	0/0	0.0
Total		**184/4172**	**4.4**	**169/4231**	**4.0**	**49/714**	**6.9**

B							
Risk group (score)		IESG development		IESG validation		Heidelberg data	
		n (%)	Deceased	n (%)	Deceased	n (%)	Deceased
Very high risk (≤ -5)		93 (2.2%)	18.3%	91 (2.2%)	14.1%	7 (1%)	14.3%
High risk (-3;-4)		378 (9.1%)	9.0%	370 (8.7%)	8.4%	20 (2.8%)	20.0%
Medium risk (-1;-2)		1308 (31.4%)	5.8%	1246 (29.5%)	5.2%	125 (17.5%)	10.4%
Lowrisk (0)		1058 (25.4%	3.0%	1173 (27.7%)	2.6%	190 (26.6%)	7.4%
Very lowrisk (≥ 1)		1335 (31.9%)	1.9%	1350 (31.9%)	2.1%	372 (52.1%)	4.6%
Total		**4172**	**4.4%**	**4231**	**4.0%**	**714**	**6.9%**

Abbreviation: IESG: International Esodata Study Group

### Steyerberg score

Attributing the respective points according to the risk factors of the Steyerberg score (Table 3A), point scores ranged between − 3 and 3 in our patient collective (Table 4A). Forty-one patients had two comorbidities, with cardiovascular disease and diabetes being the most common combination.

The 30-day mortality in our cohort was 3.4%. Table 4A shows the 30-day mortality for each group.

### Fuchs score

After calculating points according to the relevant parameters (Table 3B), patients in our cohort reached scores between 0 and 10 compared to 0 and 16 in the Fuchs cohort (Table 4B). According to Fuchs et al., 675 patients (94.5%) had low (0–7 points), and 39 (5.5%) had a high risk (8–16 points) for in-hospital mortality. The in-hospital mortality was 6.07% in the low-risk group and at 17.95% in the high-risk group, resulting in an overall mortality rate of 6.72%.

**Table 3:** Score system and results for (A) Steyerberg and Heidelberg data, (B) Fuchs and Heidelberg data

A						
		Points	Steyerberg data		Heidelberg data	
			n	%	n	%
Age	< 50	-1	368	10.3	90	12.6
	50–65	0	493	13.7	386	54.1
	> 65	1	2731	76.0	238	33.3
Comorbidities	CVD	1	416	12.9	110	15.4
	Pulmonary	1	369	11.47	46	6.4
	Diabetes	1	251	7.8	93	13.0
	Renal	1	11	0.5	17	2.3
	Hepatic	1	< 12	< 0.6	5	0.7
Neoadjuvant treatment	RTX	1.5	365	10.2	7	1.0
	RCTX	1	284	7.9	161	22.6
Hospital volume	≤ 1	0	675	33.0	0	0
	1.1–2.5	-0.5	527	25.8	0	0
	≥ 2.6	-1.5	2041	41.1	578	80.9
	≥ 50	-2	0	0	136	19.1

B						
		Points	Fuchs data		Heidelberg data	
			n	%	n	%
Age	< 45	0	1616	6.8	34	4.8
	45–64	1	10489	44.2	404	56.6
	65–74	2	7200	30.3	218	30.5
	> 75	3	4428	18.7	58	8.1
Comorbidities	CVD	1	2506	10.6	110	15.4
	Pulmonary	1	3505	14.8	46	6.4
	Renal	2	414	1.7	17	2.4
	Hepatic	3	584	2.5	5	0.7
Tumor pathology	Adenocarcinoma	0	15978	67.2	514	72.0
	Squamous cell carcinoma	1	7799	32.8	200	28.0
Abdominal approach	Laparoscopic	0	741	3.1	90	12.6
	Open	2	23162	96.9	624	87.4
Hospital volume	> 50	0	3800	16.0	87	12.2
	15–50	2	6627	27.9	627	87.8
	< 15	3	13324	56.1	0	0

Abbreviation: RTX: Radiotherapy; RCTX: Radiochemotherapy CVD: Cardiovascular disease

**Table 4:** Results according to risk groups a: Steyerberg and Heidelberg data, B: Fuchs and Heidelberg data

Points	Expected mortality	Steyerberg Score		Heidelberg data	
		Deceased/total	%	Deceased/total	%
-3	3.3%			1/65	1.5
-2	4.5%			8/334	2.4
-1	6.1%			9/236	3.8
0	8.2%			1/15	6.7
1	11.0%			4/55	7.3
2	14.6%			1/8	12.5
3	19.0%			0/1	0.0
**Total**		**291/3592**	**8.1**	**24/714**	**3.4**

Points	Expected mortality	Fuchs score		Heidelberg data	
		Deceased/total	%	Deceased/total	%
0	1.1%		0	0/1	0.0
1	1.4%		1.3	0/10	0.0
2	1.8%		0.6	0/21	0.0
3	2.4%		2.1	2/51	3.9
4	3.2%		3.0	1/53	1.9
5	4.2%		4.4	8/199	4.0
6	5.5%		5.9	15/218	6.9
7	7.3%		7.6	15/122	12.3
8	9.6%		10.5	5/32	15.6
9	12.6%		15.0	2/6	33.3
10	16.6%		17.3	0/1	0.0
11	22.0%		18.2	-	-
12	28.9%		23.3	-	-
13	38.0%		34.5	-	-
14	50.1%		33.3	-	-
15	66.0%		100	-	-
16	86.9%		0	-	-
**Total**		**1829/23751**	**7.7**	**48/714**	**6.7**

### Validation of the scores

**Fig. 1:**
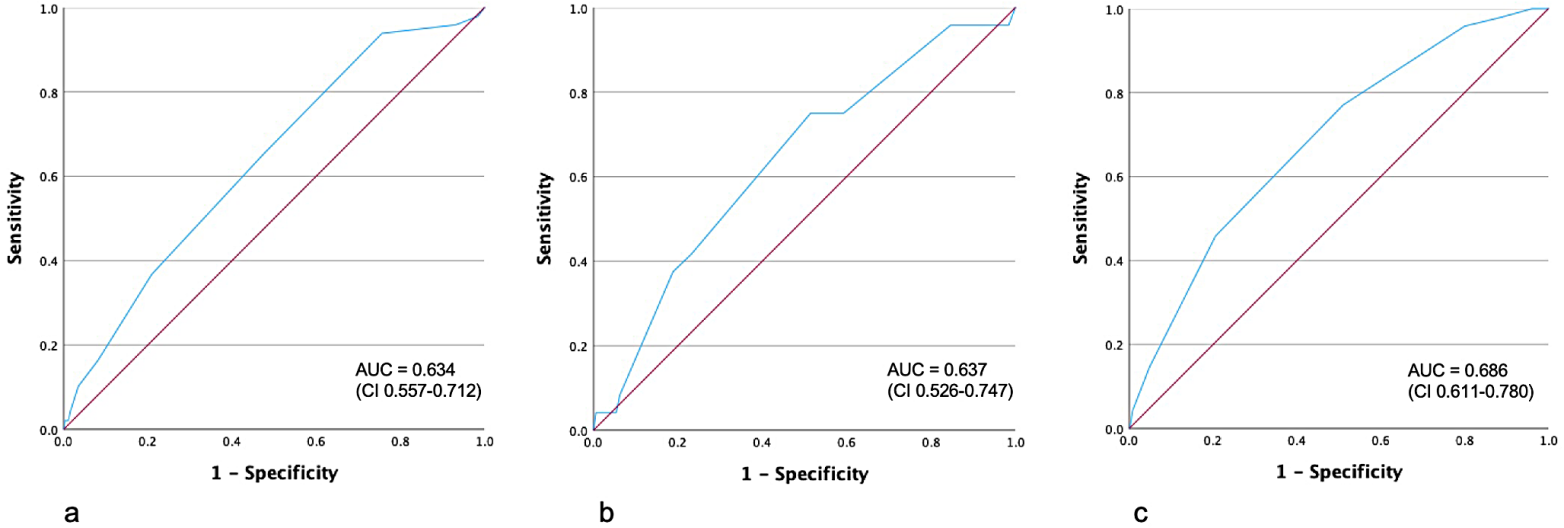
ROC curve for (**a**) IESG score, 90-day-mortality; (**b**) Steyerberg score, 30-day-mortality; (**c**) Fuchs score, In-hospital mortality

In terms of diagnostic performance, the area under the ROC curve (AUC) was 0.634 (95%CI: 0.557–0.712) for the IESG risk prediction model, 0.637 (95%CI: 0.526–0.747) for the Steyerberg score, and 0.686 (95%CI: 0.611–0.760) for the Fuchs risk scale. Results are shown in Fig. 1a-c.

## Discussion

Preoperative risk stratification is an essential tool for identifying high-risk patients. A 2020 review found 26 preoperative predictive scores of postoperative mortality, such as the Preoperative Score to Predict Postoperative Mortality, the American Society of Anesthesiologists classification system (ASA), or the Charlson comorbidity index (Charlson et al. 1987; Le Manach et al. 2016; Saklad 1941).

In 2021, the IESG developed a new risk score to predict 90-day mortality after esophagectomy (D’Journo et al. 2021). Before introducing a risk prediction model to clinical use, it should be validated on patient populations. To our knowledge, this is the first study to validate the IESG score in a single-center high-volume center which has not contributed toIESG data.

Steyerberg et al. reported in 2006 a predictive score including eight characteristics related to the outcome after esophagectomy (Steyerberg et al. 2006). A key limitation of this score is that it relies on patient data from the 1990s. As morbidity and mortality of esophagectomy have dramatically changed over the past three decades, the prognostic value of the Steyerberg score might be limited. However, the AUC of the Steyerberg score in our cohort was 0.637, confirming the initial study’s results (AUC 0.56–0.70). Compared to the original publication of the Steyerberg score, our 30-day mortality was lower (3.0% compared to 4%,7%,10%, and 11%). The most likely explanation for this difference is the abovementioned improvement in perioperative treatment. Furthermore, Steyerberg’s score is based upon SEERs (Surveillance, Epidemiology, and End Result) data, which included lacks granularity of institutional databases and therefore may have missed some relevant predictive factors including ASA (American Society of Anesthesiologists) classification.

The in-hospital mortality score by Fuchs used six patient factors (age, cardiovascular, pulmonary, renal and hepatic comorbidity and tumor pathology) and two hospital factors (operation volume/year, abdominal approach) to calculate the mortality (Fuchs et al. 2017). The AUC for the Fuchs score was 0.686, which also agrees well with the score. The in-hospital mortality rate in our hospital was 6.7% compared to 7.7%, as calculated by the Fuchs score.

The Fuchs score’s main limitation is the reliance on a nationwide database, including data on discharge records. These databases rely on accurate data but may lack details on complex cases, like preoperative functional or nutritional status or neoadjuvant treatment.

Even though considerable differences between the cohorts of the original publication of the IESG score and our cohort exist, predictive values were similar with 0.634 compated to 0.68 in development and 0.64 in the validation group. Almost 60% of the patients in the IESG cohort had a minimally invasive operation, whereas in our cohort over 20 years starting in 2002, a majority received an open operation (87.4%). In the IESG cohort on the other hand the investigation period was relatively short with just four years. Although the operation technique is not part of the risk stratification for IESG, this finding shows similar performance in different settings. A limitation pertinent to the IESG score is its derivation from the Esodata database, which was not designed for this specific research questions and lacks the capacity for in-depth analysis or the consideration of additional variables.

However, preoperative risk stratification should be practical and reliable. Complex scores with too many parameters are less suitable for clinical use. Parameters that need to be calculated or analyzed may need to be more practical for the clinical routine. The IESG score includes ten parameters compared to eight factors included in the Steyerberg and eight factors in the Fuchs score. Inclusion of more parameters should naturally result in a more sophisticated model yielding a more accurate risk prediction. However, in our collective all scores revealed rather low predictive power irrespective of the number of parameters included. The parameters used in the IESG score all seem reasonable and are simple to collect. Yet inclusion of ten parameters each with various numerical values results in a cumbersome calculation process which seemed slightly too complex for the clinical implementation of this score in the past. Yet, rapid advancements in artificial intelligence (AI) and related technologies have significantly expanded the possibilities for complex risk prediction models. While modern tools could manage a larger number of parameters more efficiently, practical challenges currently faced in routine clinical workflows, such as time constraints, user training, and accessibility in resource-limited settings still have to be considered. Future risk prediction models should aim to leverage the transformative potential of these innovations.

The three models differed in the included comorbidities for calculating the score. Whereas Fuchs and Steyerberg found that cardiovascular, pulmonary, renal, and hepatic comorbidities had an impact, the grade of the impact differed. For example, Steyerberg gave every comorbidity one point, whereas Fuchs’s score shows more severity for renal and especially hepatic comorbidities. The IESG score, on the other hand, just gave a higher score for people with moderate or severe liver disease and did not include pulmonary or renal comorbidities but connective tissue disease and peripheral vascular disease. All investigated scores carry the risk that comorbidities are underestimated and not entirely diagnosed at the time of the risk assessment. Combining all published studies on this topic, a meta-analysis showed that an age ≥ 70 and cardiac and renal comorbidities significantly impacted the 30-day- and in-hospital mortality. In contrast, the meta-analysis did not show a significant impact of a BMI < 18.5 and pulmonary comorbidities on the 30-day- and in-hospital mortality. The alcohol intake that was also a significant prognostic factor for a 30-day- and in-hospital mortality (OR 3.1 (95%CI 2.26–4.25) was not included in any of the three predictive scores (van Kooten et al. 2022).

Another important parameter in all three scores is hospital volume. High-risk procedures such as esophagectomy should be performed in high-volume centers to reduce morbidity and mortality (Markar et al. 2012; Metzger et al. 2004).

Our study has some limitations. It is a retrospective single-center evaluation with a smaller sample size than the original publications (IESG 8403, Fuchs 23751, Steyerberg 3592 patients). Another limitation of this study is the long study period from 2002 till 2021. As noted in the introduction, substantial changes have occurred in surgical techniques, intensive care, and complication management. These advancements have likely contributed to improved outcomes in recent years. The long study period could also explain the higher mortality rates observed in our cohort’s low- and very low-risk groups compared to the IESG cohort. When analyzing only patients treated from 2015 to 2021, the mortality rates decrease to 3.2% for the low-risk group and 1.3% for the very low-risk group, which are consistent with the results reported by the IESG.

Even when postoperative mortality could be reduced over the years, complication rates of up to 22–70% are mainly caused by respiratory complications like pneumonia with respiratory failure, arrhythmia, anastomotic leakage, and wound infection (Atkins et al. 2004; McLoughlin et al. 2013; Raymond et al. 2016). Results of the IESG score showed that this kind of complications occur more often in very high and high-risk patients (D’Journo et al. 2021).

How can we apply high-risk patient identification in practice? First, surgery indications for high-risk patients should be critically reconsidered, especially if definitive radiochemotherapy is a viable alternative. If neoadjuvant radiochemotherapy shows a good response, surgery with its risks might be postponed in favor of regular follow-up in selected cases. However, with an AUC of < 0.7 in all scores, prediction is unreliable and therefore the scores show insufficient performance to guide these important treatment decisions.

Second, procedures such as prophylactic endoluminal vacuum therapy should be considered in patients with increased risk profile. Studies in this regard currently need to be improved. A systematic review of prophylactic vacuum therapy identified four case series for patients undergoing esophagectomy for cancer treatment (Adamenko et al. 2022). The biggest case study with 67 patients, whereas 57 patients were identified as high-risk patients (ASA > 2, BMI > 29, WHO/ECOG score > 1, age > 65 years), was done after minimal invasive Ivor-Lewis procedure. The morbidity rate was 40% for minor and 15% for major morbidity. 73% of the patients showed an uneventful healing of anastomosis. The anastomosis leakage rate was 7.5% (Müller et al. 2021). Thus, prophylactic endoluminal vacuum therapy could reduce the rate of anastomosis leakage and therefore, may also reduce morbidity and mortality rates, but high-evidence is lacking. As it might be a potential option for high-risk patients the potential drawbacks of prophylactic endosponge therapy must be considered. One notable concern is its possible interference with established enhanced recovery after surgery (ERAS) or fast-track programs, which have been consistently shown to improve postoperative outcomes (Huang et al. 2024). This conflict underlines the need for a balanced approach carefully evaluating risks and benefits of prophylactic endosponge therapy. Future research should focus on clarifying its efficacy and determining its role in the context of high-risk patients and ERAS protocols.

Last but not least, high-risk patients should be monitored more intensively after surgery to identify and treat complications early, in order to reduce perioperative morbidity and mortality by their timely treatment.

In conclusion, the existing scoring systems provide a possibility for individual preoperative risk stratification, especially to identify high-risk patients. However, strategies for identifying these patients and their application in everyday clinical life currently need to be improved due to their low predictive ability.

## Data Availability

No datasets were generated or analysed during the current study.
